# Distinguishing sterile inflammation from graft infection

**DOI:** 10.1186/s13019-024-02504-5

**Published:** 2024-01-23

**Authors:** Atsuyuki Mitsuishi, Yujiro Miura, Yu Arakawa, Tatsuya Noguchi

**Affiliations:** 1 Department of Cardiovascular Surgery, School of Medicine, Kochi University, 185-1, Kohasu, Nankoku-shi, Okohmachi, Kochi Prefecture, 783-8505 Japan; 2 Department of Cardiovascular Surgery, Kochi Medical School, 185-1, Kohasu, Nankoku-shi, Okohcho, Kochi Prefecture, 783-8505 Japan; 3 Department of Clinical Infectious Diseases, Kochi Medical School, 185-1, Kohasu, Nankoku-shi, Okohcho, Kochi Prefecture, 783-8505 Japan; 4 Department of Cardiology and Geriatrics, Kochi Medical School Hospital, 185-1, Kohasu, Nankoku-shi, Okohcho, Kochi Prefecture, 783-8505 Japan

**Keywords:** Sterile, Inflammation, Non-bacterial, Graft infection, Peri-graft, Fluid collection

## Abstract

We describe the case of a 68-year-old man who underwent ascending aortic replacement and thoracic endovascular aortic repair. Four years later, the patient developed neck pain on the right side and chest computed tomography showed expansion of fluid in the mediastinum which had extended to the neck. Echocardiography revealed advanced severity of aortic regurgitation and decreased ejection fraction. Given the progression of aortic regurgitation, decreased cardiac function, and rapidly expanding fluid accumulation causing neck pain, reoperation was indicated. All microbiological test including polymerase chain reaction were negative indicating absence of any infection. The patient is being followed-up without antibiotics and CT has not shown peri-graft fluid 2 years postoperatively. Since infection cannot be excluded completely, it is important to assess the condition with selective medium, extended culture periods, genetic testing, and consultations with microbiology laboratories when normal culture tests for general bacteria, and fungi are negative which can help avoid drug-resistant bacteria count, elevated medical costs, and drug side effects due to the improper use of antibiotics through proper diagnosis.

## Introduction

### Case presentation

A 68-year-old man underwent partial arch replacement with brachiocephalic artery reconstruction for Stanford type A dissection at our hospital 4 years ago and in the same month, zone 1 two-debranched thoracic endovascular aortic repair (2d-TEVAR) (C-TAG; 3120 − 2615, GORE) with an axillo-axillary and axillo-left common carotid bypass was performed for enlargement of the false lumen at the distal arch (50 mm). He was not immunocompromised and was not being treated with any immunosuppression agents and he never had any sternal infection since partial arch replacement and 2d-TEVAR were performed.

Annual follow-up computed tomography (CT) conducted 1 month before the second surgery, revealed fluid collection around the ascending aorta graft without any symptom. No fluid surrounding the neck was observed at the time.

**Fig. 1:**
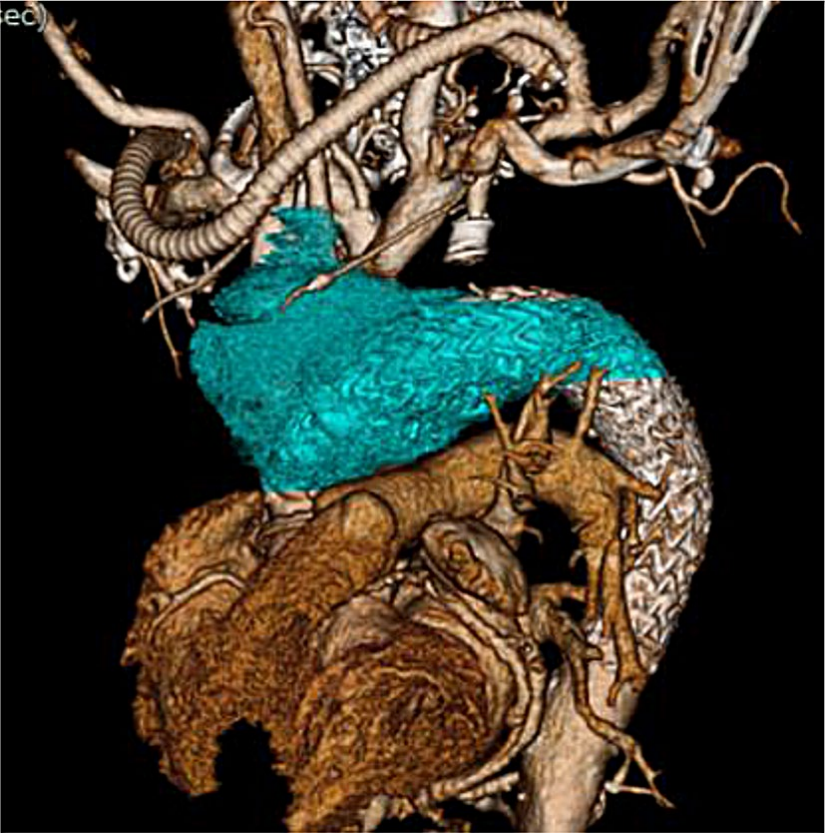
The CT value of peri-graft fluid collection (Blue color) was 30–40 HU

**Fig. 2:**
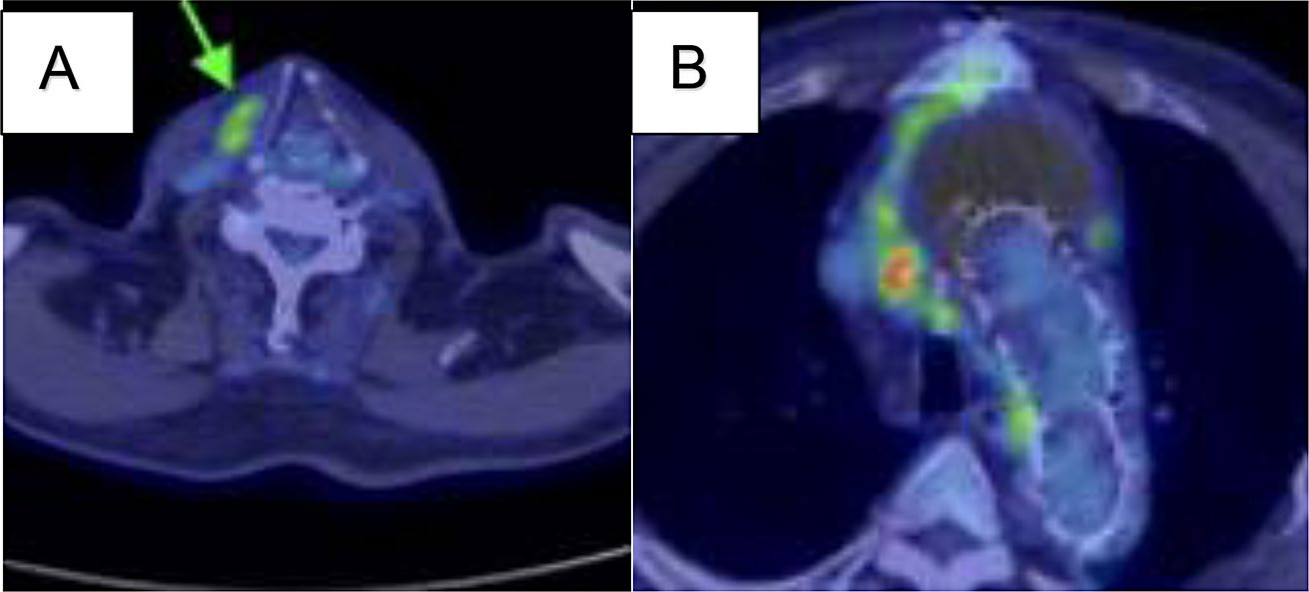
**A**, **B** (18)F-fluorodeoxyglucose ((18)F-FDG) positron-emission tomography (PET) before surgery. **A** The fluid in the mediastinum had expanded and extended to the right neck side (green arrow). **B** The PET confirmed 14.2 standardized uptake values max (SUV max) around prosthetic and stent graft

Two weeks before the second surgery, the patient complained of neck pain in the right side, and plain CT revealed that the fluid in the mediastinum had expanded and extended to the right neck side (Fig. 1). The patient developed fever and was hospitalised. Blood test showed white blood cell (WBC) 10.2 × 1000/µL (normal value, 3.3–8.6 × 1000/µL); neutrocytes 86.2%(normal value, 41.2–74.7%), lymphocyte 5.9% (normal value; 21.2–51%), monocytes 7.5% (normal value, 3.1–8.0%), eosinophiles 0.1% (normal value, 0.2–8.4%), basophils 0.3% (normal value, 0.2–1.8%), and C-reactive protein 10.75 mg/dL (normal value, < 0.14 mg/dL). Following two sets of blood culture, antibiotic (ampicillin 3 g/12 h) was initiated, following which the fever subsided. ^18^F-fluorodeoxyglucose (^18^F-FDG) positron-emission tomography (PET) confirmed high accumulation (standardized uptake values max, SUV max) around the fluid (Fig. 2A, B). PET confirmed 14.2 SUV max around prosthetic and stent graft. Graft infection was suspected given the improvement in fever after administration of antibiotics, improvement in blood test findings, and accumulation noted on PET. CT-guided needle aspiration was conducted. Approximately 150 mL of yellow-white and highly viscous fluid was collected, showing an abundance of neutrophils, which was suggestive of infection upon microscopic examination but the culture was negative. Echocardiography revealed advanced severity of aortic regurgitation and decreased ejection fraction. Given the progression of aortic regurgitation, decreased cardiac function, and rapidly expanding fluid retention causing neck pain, surgery was indicated.

**Fig. 3:**
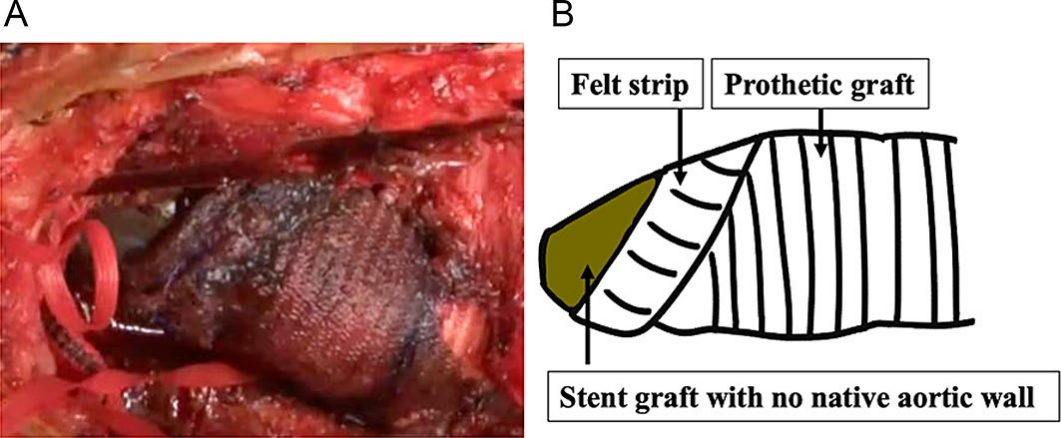
**A**, **B** Operative image (**A**) and schematic (**B**)

Thorough a median re-sternotomy, avoiding the subclavian artery graft bypass, cardio-pulmonary bypass was established with femoral artery and vein. Abscess-like fluid erupted in the mediastinum; which was cultured. The stent graft was exposed with no native aortic wall observed (Fig. 3A, B). The stent was placed in zone 1 and no migration was observed. Yellowish-white and highly viscous fluid retained around prosthetic and stent grafts. Under 19.6 °C at bladder, and 19.6 °C tympanic temperature, retrograde cardioplegia which comprised 26 mEq potassium chloride added to 500 mL St. Thomas II solution was administered every 20 min. Selective cerebral perfusion from prosthetic graft connected with brachiocephalic artery was started at 700 mL/min.

**Fig. 4:**
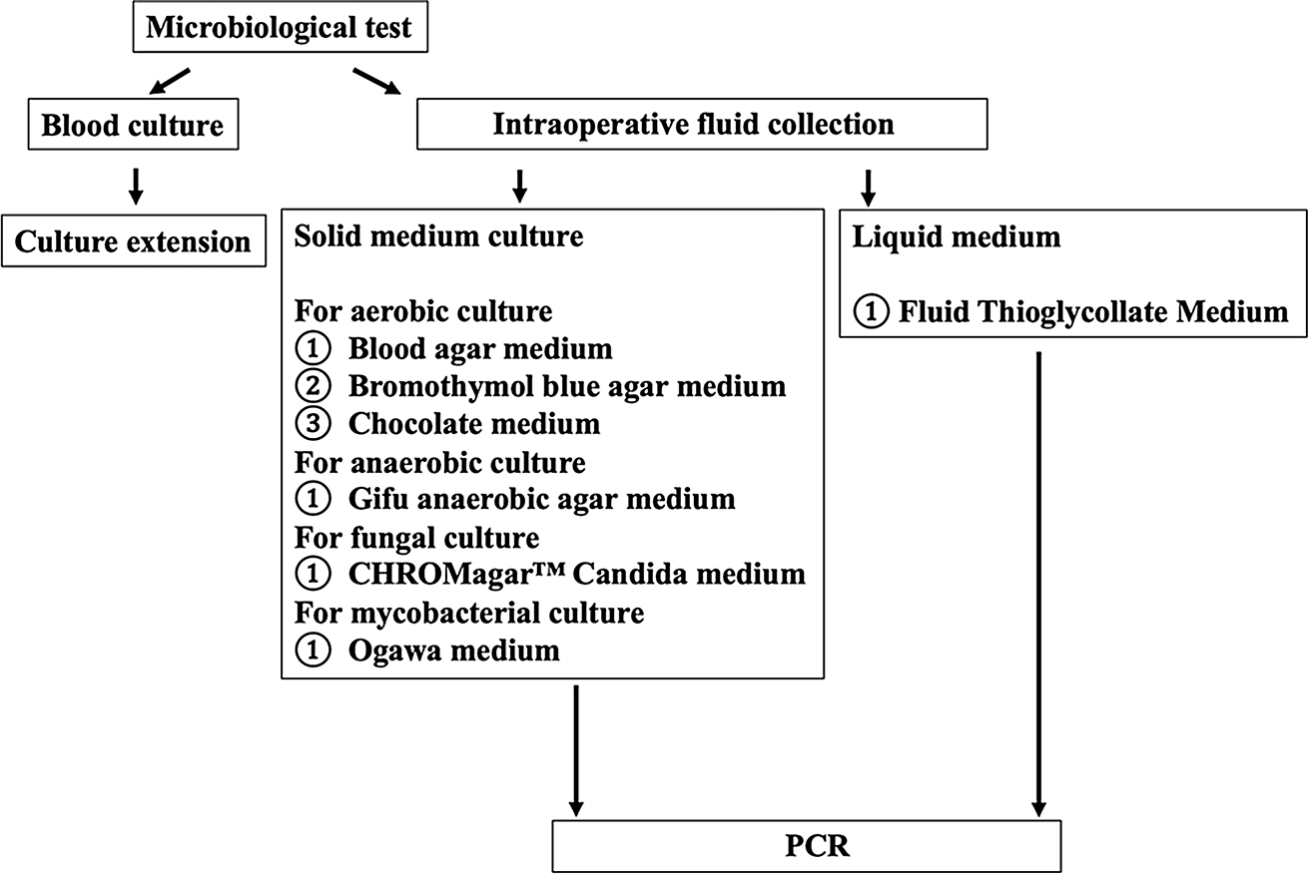
Algorism of microbiological test. PCR; Polymerase chain reaction

The proximal and distal anastomosis was highly adhered. We attempted to remove all artificial materials as much as possible due to the risk of infection, but the patient had a high degree of adhesion, and considering surgical invasiveness, we did not perform extended surgery. The aortic valve was tricuspid without prolapse, and the aortic regurgitation (AR) was thought to be due to annular dilatation. Although aortic annulus was 25 mm, due to a felt strip inside the proximal anastomosis, and the narrow sinotubular junction, we managed to put a 21-mm bioprosthetic valve (INSPIRIS RESILIA aortic valve, Edowards, California, United States of America) on the supra-annular position. The aortotomy was closed in two layers. Haemostasis was confirmed in the pericardium, and the omentum was collected. The area around the grafts was washed with lavage fluid, and omental filling was conducted, whereupon the surgery was completed. Cardiac arrest time was 115 min and cardiopulmonary bypass time was 254 min. After consultation with the infectious disease department and microbiology laboratory, additional cultures from all preoperative and intraoperative fluid collections were performed in solid and liquid medium for enrichment culture (Fig. 4). Two sets of blood samples were taken prior to antimicrobial administration and cultured using BACT/ALERT 3D (BioMérieux, Marcy-l’Étoile, France) for seven days. However, no bacteria was detected. The preoperative blood cultures were extended for 4 weeks, but they were still negative. All these cultures were negative which was also confirmed using polymerase chain reaction (PCR) with 16srRNA for bacteria and 18srRNA for fungi.

Post-operative antibiotic, ampicillin 3 g/12 h for 11 days after the second operation was discontinued after negative PCR results. The patient is being followed-up without antibiotics and CT has not shown peri-graft fluid 2 years postoperatively.

## Discussion

It is often difficult to distinguish non-infectious inflammatory response from infection in peri-graft fluid collection. The diagnosis of graft infection is usually based on clinical findings supported by the clinical course and radiological and microbiological investigations.

In clinical course, non-infectious persistent fluid collection or soft-tissue attenuation was observed for up to 3 months post-operatively [1]. In some cases, peri-graft fluid may be seen for up to 1 year without infection [2].

On CT, abscesses may present as low-attenuation masses, exhibiting rim enhancement following contrast agent injection [3–7]. Other findings suggestive of infection include air bubbles within the abscess cavity and adjacent soft tissue [3, 4, 6, 8–10].

**Fig. 5:**
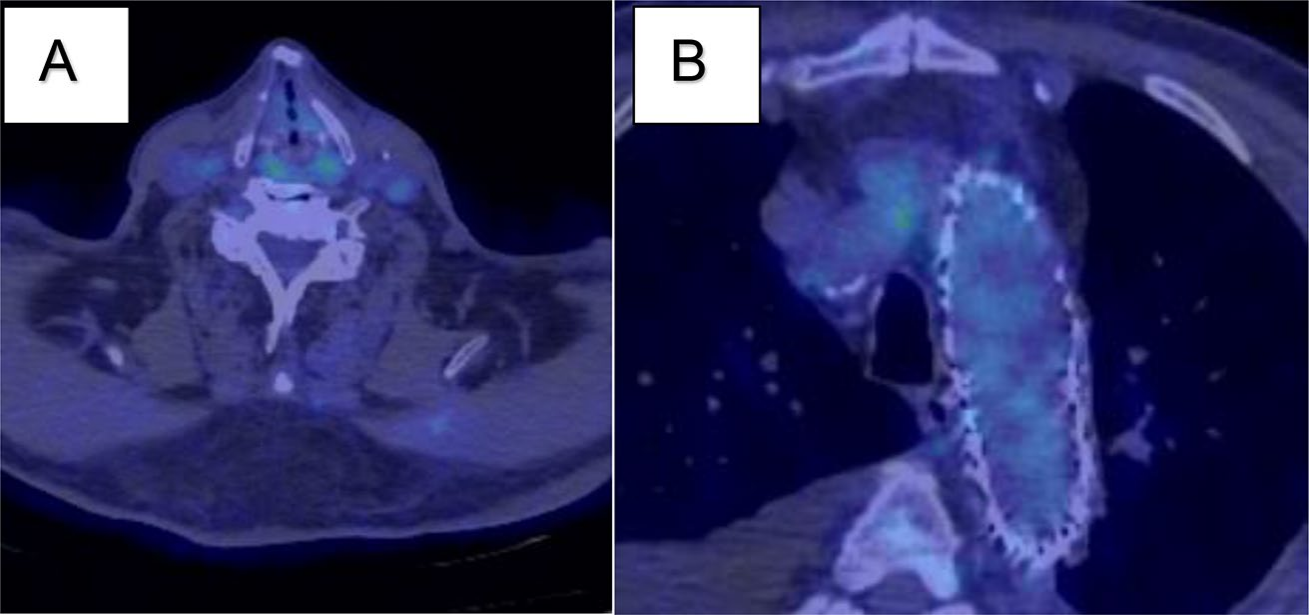
**A**, **B** (18)F-fluorodeoxyglucose ((18)F-FDG) positron-emission tomography (PET) 2 years after surgery. **A**, **B** The PET confirmed decreased to 3.1 standardized uptake values max (SUV max) around prosthetic and stent graft

Regarding CT values and SUV max, it has been reported that an abscess is > 25 Hounsfield unit (HU) and a seroma is < 25 HU [10] and some authors have reports 6.3 SUV as a good cut-off value for differential diagnosis of inflammation and infection [11], even though there are no general criteria published for these two processes. It has also reported that SUV max cut off values could serve as an interesting monitoring tool for the response to antibiotic treatment [12]. In this case, ^18^F-FDG PET-CT turned to be negative 2 years after second surgery (Fig. 5A, B). The PET confirmed 3.1 SUV around prosthetic and stent graft. Regarding blood culture, it can be false negative, or the same bacteria as graft infection may not be detected, CT-guided needle aspiration for culture has been proposed to obtain fluid collection around the graft [13]. In addition, bacteria may not be detected even though a large amount of specimens are collected.

Possibilities include attenuated bacteria, such as *Staphylococcus epidermidis*, in which a large amount of WBC is observed but no microorganisms are detected [14], and the culture becomes negative when antibiotics administration precedes. In that case, the sensitivity can be increased by extending the culture period [15] or by transferring to enriched medium. Furthermore, PCR is more sensitive and contributes to diagnosis of graft infection in patients with culture-negative infections [16].

We conducted CT-guided needle aspiration for peri-graft fluid collection around the prosthetic and stent grafts and collected approximately 70 cc of a highly viscous, yellowish-white WBC specimen. The culture showed negative finding even in a liquid medium for enrichment culture, and PCR was also confirmed as negative. We also suspected seromas, accompanied by a capsule, which represent most common non-infectious cases [17–19]. However, no such capsule was observed during the re-operation. The second time, the stentsgraft was exposed during surgery, and no native blood vessels were observed. It is thought that this might be related to fluid collection around stent graft in this study. It is reported that stent-graft placement causes a thinning of blood vessels. The aortic wall is nourished by vasa vasorum and endoluminal diffusion [19–21]; in TEVAR, an endovascular stent-graft covers the vessel luminal wall, disrupting both trophic pathways. As a result, necrosis of the intima [22], media [23], and adventitia [24] has been reported. In our patient, the cause of fluid collection around the vascular prosthesis was compression of the vaso-vasorum due to the use of an oversized stent and excessive stress being applied, resulting in aortic ischaemia and inflammatory changes, with inflammation potentially forming a non-infectious abscess. This case report has some limitations. First, the infection may be controlled only by the omentum although it is rare to have no recurrence of the infection without long-term antibiotic use and removal of prosthetic or stent graft. Second, the patient has been followed-up only for 2.5 years without antibiotics. Since peri-graft fluid collection occurred 2 years after first surgery, there is a possibility that symptoms will appear in the future if they are the same microbes.

We cannot conclude that extending the culture period, submitting additional enriched culture and PCR can exclude the infection completely, but it may help in decision for discontinuation of antibiotics. Third, necrosis of the wall of the aorta has been attributed to pressure effect and ischemia. In such scenario, obliteration by infection cannot be ruled out.

## Conclusion

We report a case of rapid peri-graft fluid accumulation 2 years after 2d-TEVAR. When graft infection is suspected in condition where normal culture tests for general bacteria and fungi are negative, we emphasise the importance of medium selection, additional medium orders, extension of culture, genetic testing, and communication with microbiology laboratories.

## Data Availability

All data generated or analyzed during this study are included in this published article.
